# Growth Restriction and Systemic Immune Development in Preterm Piglets

**DOI:** 10.3389/fimmu.2019.02402

**Published:** 2019-10-10

**Authors:** Ole Bæk, Per Torp Sangild, Thomas Thymann, Duc Ninh Nguyen

**Affiliations:** Section for Comparative Pediatrics and Nutrition, Faculty of Health and Medical Sciences, University of Copenhagen, Frederiksberg, Denmark

**Keywords:** growth restriction, intrauterine, extrauterine, immune system, development, immunity, preterm, infant

## Abstract

**Background:** Many preterm infants are born with growth restriction (GR) following maternal or fetal complications before birth. Such infants may continue to grow slowly after birth, regardless of birth weight (BW), due to morbidities related to their immature organs. Severe GR increases the susceptibility to infections, but it is not clear if this is a consequence of impaired systemic immunity or other factors, such as prolonged hospital stay or poor mucosal barrier function. Using preterm pigs as models for preterm infants, we hypothesized that moderate GR, exerting limited clinical effects, does not influence systemic immune development.

**Methods:** Preterm pigs were delivered by cesarean section and fed bovine milk diets until 19 d. Piglets with fetal growth restriction (F-GR, the lowest 25% of BW, *n* = 27, excluding those with BW <350 g) and postnatal growth restriction (P-GR, the lowest 25% of postnatal growth rate, *n* = 24) were compared with their corresponding controls (F-CON, *n* = 92, and P-CON, *n* = 85, respectively). Organ weights were determined and blood collected for assessment of clinical status (blood chemistry and hematology). For a subgroup (*n* = 58), in depth analyses of neutrophil function, T cell counts, plasma cytokine levels, and leucocyte gene expression were performed.

**Results:** For F-GR pigs, adrenal gland weight was increased and bone mineral content decreased at 19 d. Total leucocyte levels were lower at birth and interleukin-10 levels increased at d 8–10. In P-GR pigs, total leucocyte, neutrophil, monocyte, and eosinophil counts along with helper T cell fractions were elevated at 8–19 d of age, while the fraction of neutrophils with phagocytic capacity was reduced. Diarrhea and all remaining organ weights, blood chemistry, and immune variables were not affected by F-GR or P-GR.

**Conclusion:** Moderate GR before and after preterm birth has limited effect on systemic immune development in preterm pigs, despite marginal effects on immune cell populations, adrenocortical function, and body composition. Similar responses may be observed for preterm infants with moderate fetal and postnatal growth restriction.

## Background

Preterm infants (<37 weeks of gestation) have an increased risk of developing life-threatening infections and up to 40% develop sepsis in the neonatal period (1). This may be related to an underdeveloped immune system, inadequate transfer of maternal antibodies, but also to iatrogenic interventions, such as mechanical ventilation and vascular catheterization (1, 2). Antenatal factors, leading to slow fetal growth, may also play a role in increasing the risk of postnatal infection (3). Severely impeded fetal growth, resulting in intra uterine growth restriction (IUGR, defined as <10% of the body weight percentile), is a consequence of placental insufficiency or medical conditions, such as preeclampsia, uterine complications, or maternal/fetal infections (4). These infants show reduced leukocyte, lymphocyte, and neutrophil counts at birth (5, 6) and leukocytes exhibited reduced capacity to be activated by lipopolysaccharide following *ex vivo* whole blood challenge (3). Likewise, newborn infants show higher tolerable bacterial loads than adults during bacteremia, possibly as a result of energy constraints (7), which may be exacerbated after IUGR. Together this may be a cause for their increased risk of neonatal sepsis and later mortality, compared with infants born with a normal body weight for their gestational age (1, 8–10). IUGR infants have also been shown to have reduced thymus size at birth, a prognostic factor for later sepsis risk (11, 12). Adolescents born IUGR have lower levels of plasma thymopoietin (13), indicating long term effects of IUGR on thymus function, while any IUGR related reduction in plasma immunoglobulin levels at birth may disappear within the first year (14). Despite these results, the immune effects of different degrees of fetal growth restriction after preterm birth remain poorly understood. Even without IUGR, preterm birth is associated with low blood cell counts and immature blood cell functions (2), and the “double hit” of IUGR and preterm birth may either increase or decrease the immune deficits documented for IUGR infants born at full term.

In addition to poor prenatal growth, a major proportion of preterm infants experience postnatal growth restriction (15). After birth, preterm infants have higher nutritional requirements than term infants and have a high risk of feeding intolerance due to an immature gut, leading to difficulties in achieving growth rates that resemble those *in utero* (15, 16). Alleviating or preventing this extra uterine growth restriction (EUGR) may be critical for later neurodevelopment (17), but it is not clear if EUGR is associated with defects in other critical functions, such as immunity. Children have higher baseline levels of inflammatory cytokines, if exposed to EUGR in infancy (18), and malnutrition induced EUGR may affect immunity in children in low-income countries (19). For preterm infants, fortification of mother's own milk may be required to provide adequate amounts of nutrients, but even with adequate nutrient intake, preterm infants may experience slow growth, indicating that multiple factors lead to EUGR (15), not only low nutrient intake, low birth weight, and shortened gestation (20, 21).

Studies in full term piglets have contributed important information about the postnatal consequences of IUGR on physiological functions, including immunity (22–25). Much less is known for preterm pigs or infants, and especially for more moderate growth restriction before or after birth. The immature status of such newborns may render them more or less susceptible to the consequences (metabolic, endocrinological, inflammatory, or other) of moderate growth restriction. Using preterm pigs as a model for preterm infants, we hypothesized that moderate fetal and postnatal growth restriction, represented by individuals with the lowest 25% birth weight or postnatal growth rates, and excluding any pigs with extremely low birth weight, would not show deficient organ growth or immune development, relative to remaining litter-mate controls. Growth restricted pigs were selected from a cohort of 7 l of preterm pigs reared for 19 days for recording of body composition, hematological, biochemical, and immune parameters.

## Methods

### Animals and Experimental Design

Using three separate animal experiments with similar design, we identified a cohort of preterm piglets to investigate the impact of fetal and postnatal growth restriction. The experiments were all performed in accordance to the principals of the Basel Declaration and approved by the Danish National Committee of Animal Experimentation (2009/561-1731). A total of 125 piglets from 7 litters (Landrace × Yorkshire × Duroc, Gadbjerg, Denmark) were born prematurely by cesarean section at day 106 (90% gestation, term at 117 ± 2 days). Immediately after birth, piglets were individually housed in heated incubators (37–38°C) and resuscitated with mechanical ventilation, if required. Four piglets died of respiratory failure before randomization, leaving 121 (54% male) piglets for the cohort study. While still anesthetized from the cesarean section, each animal was prepared with an orogastric catheter for enteral feeding and an umbilical arterial catheter for parenteral nutrition and blood sampling. All piglets were passively immunized by systemic infusion of maternal plasma (total of 25 mL/kg, in three boluses within the first 24 h).

Piglets were fed increasing amounts (16–180 ml/kg/day) of bovine milk fortified with bovine colostrum (Biofiber Damino, Gesten, Denmark), whey protein concentrate and/or human milk oligosaccharide fractions (all products from Arla foods ingredients, Viby, Denmark) until postnatal day 19. All pigs were fed the same relative amounts of diet according to their body weight. The macronutrient levels in enteral diets were 33–43 g/L of carbohydrate, 38–52 g/L of fat, and 27–55 g/L of protein, resulting in 2.6–3.6 MJ/L. An overview of the different dietary regimens are shown in Table 1. To prevent diarrhea all animals were given oral antibiotics within the first 10 days of life. A combination of antibiotics were used consisting of: amoxicillin with clavulanic acid (Bioclavid, Sandoz GmbH, Kundl, Austria), gentamicin (Gentocin Vet, ScanVet, Fredensborg, Denmark) and metronidazole (Flagyl, Sanofi-aventis, Hørsholm, Denmark), given as 2 doses/day on day 9 and 10 (litters 1–4) or 2 doses/day on day 1–5 (litter 5–7).

**Table 1:** Study overview.

**Study characteristics**				**Feeding volumes (ml/kg/day)**									
**Litters**	***N***	**Blood sample (days)**	Immune subgroup^*^	**Day 1**	**Day 2**	**Day 3**	**Day 4**	**Day 5**	**Day 6**	**Day 7-8**	**Day 9**	**Day 10–15**	**Day 16–19**
1	10	1^♢^, 8° & 19^□^	Yes	32	48	64	96	96	112	128	144	160	180
2	10	1^♢^, 8° & 19^□^	Yes	32	48	64	96	96	112	128	144	160	180
3	23	1^♢^, 8° & 19^□^	Yes	32	48	64	96	96	112	128	144	160	180
4	17	1^♢^, 8° & 19^□^	Yes	32	48	64	96	96	112	128	144	160	180
5	21	1^♢^, 10^□^ & 19^•^	No	16	32	48	64	96	112	128	144	160	180
6	24	1^♢^, 10^□^ & 19^•^	No	16	32	48	64	96	112	128	144	160	180
7	20	1^♢^, 10^□^ & 19^•^	No	16	32	48	64	96	112	128	144	160	180

* Blood samples used for T cell characterization, neutrophil phagocytic capacity, lymphocyte gene expression, and plasma cytokine levels.

♢ Cord blood; °, Umbilical catheter;

□ Jugular vein puncture;

• *Cardiac puncture*.

Parenteral nutrition (PN, Kabiven and Vitalipid, Fresenius Kabi, Uppsala, Sweden) was optimized to preterm piglets and contained 2.8 MJ/L of energy, 72 g/L of glucose, 31 g/L of lipid, and 22 g/L of amino acids. PN was provided from birth until day 7 (decreasing from 120 to 48 ml/kg/day) at which time PN nutrition was stopped. For litters 1–4, the PN was replaced with a saline solution to maintain the catheters until blood sampling on day 8. For litters 5–7 the umbilical catheters were removed after secession of PN on day 7. The same TPN formulation was used for all animals, regardless of dietary regimen. On day 10, piglets were moved to larger individual cages, still with adequate heating and free access to drinking water (tap water) until euthanasia on day 19.

Within each litter, fetal growth restricted preterm pigs were defined as pigs with the lowest 25% birth weight (F-GR, *n* = 27), excluding extremely growth restricted piglets below 5% of mean birth weight (corresponding to ~350 g, *n* = 2, due to mortality shortly after birth) and compared with the remaining pigs in each litter (F-CON, *n* = 92). For the evaluation of postnatal growth restriction until day 19, animals that survived at least until day 14 were included (*n* = 109). Within each litter, piglets with the 25% lowest growth rate, as in relative body weight increase per day (g/kg/day), from birth to 14 days were defined as postnatally growth restricted (P-GR, *n* = 24) and compared with the remaining pigs in each litter (P-CON, *n* = 85). Given the study design, there was an overlap between the fetal and postnatal growth restricted groups, as illustrated in Table 2.

**Table 2:** Distribution of pig numbers in groups.

	**P-GR**	**P-CON**	**Total**
F-GR	6	14	20
F-CON	18	71	89
**Total**	24	85	109

*Number of fetal growth restricted preterm pigs and controls (F-GR, F-CON) and, postnatally growth restricted preterm pigs and controls (P-GR, P-CON)*.

### Blood Sampling, Body Composition, and Tissue Collection

Blood samples (~1.5 mL) were collected at different time points, including cord blood from birth (all litters), in the morning of day 8 from umbilical catheter (litter 1–4), in the morning of day 10 from jugular vein puncture (litter 5–7) and day 19, either by jugular vein puncture, in the morning before euthanasia (litter 1–4) or by cardiac puncture at euthanasia (litter 5–7). EDTA stabilized blood samples were used for hematology, T cell subset phenotyping, analysis of neutrophil phagocytosis functions and whole blood gene expressions. EDTA stabilized plasma was used for cytokine assays and serum for biochemical analyses. On day 19, before euthanasia, animals were anesthetized and subjected to a full body dual energy X ray absorptiometry (Lunar Prodigy scanner, GE Healthcare, Little Chalfont, UK) to determine body composition. Afterwards all animals were euthanized by an intracardial injection of pentobarbital (60 mg/kg) after which the weight of all internal organs was recorded.

### Hematology, Blood Biochemistry, and Systemic Immune Parameters

Hematology and serum biochemistry were performed using an Advia 2120 hematology system and an Advia 1800 Chemistry System, respectively (Siemens Healthcare Diagnostics, Tarrytown, NY, USA). For a subgroup of 58 animals (F-GR = 11, F-CON = 47, P-GR = 11, P-CON = 39), additional evaluation of systemic immune maturation was performed. T cell characterization, neutrophil phagocytic function, leucocyte gene expression, and plasma cytokine levels were determined using EDTA stabilized blood samples from day 1, 8, and 19. For T cell subset characterization, erythrocytes from blood samples were lysed (BD lysing solution, BD Biosciences, USA) and leucocytes were washed and incubated with fluorescent antibodies against porcine CD3 (PerCP-Cy5.5-conjugated mouse anti-CD3, IgG2a isotype, BD Bioscience), CD4 (FITC-conjugated mouse anti-pig CD4, IgG2b isotype, Biorad, Copenhagen, Denmark), and CD8 (PE-conjugated mouse anti-pig CD8, IgG2a isotype, Biorad). Negative controls included PerCP-Cy5.5-conjugated mouse IgG2a isotype control (BD Bioscience), PE-conjugated mouse IgG2a negative control, and FITC-conjugated mouse IgG2b negative control antibodies (Biorad). Leukocytes were analyzed by flow cytometry using BD Accuri C6 flow cytometer (BD Biosciences, USA). Lymphocyte population was gated using the forward scatter (FSC) and side scatter (SSC) dot plots, and the lymphocyte subsets were defined as follows: T cells (CD3^+^ lymphocytes), helper T cells (CD3^+^CD4^+^CD8^−^ lymphocytes), cytotoxic T cells (CD3^+^CD4^−^CD8^+^ lymphocytes). Median fluorescent index (MFI) was used to estimate surface expression levels of CD4 and CD8.

Blood neutrophil phagocytosis function was tested by *ex vivo* whole blood stimulation with fluorescent marked *Escherichia coli* (pHrodo Red *E. coli* (560/585 nm), using bioparticles phagocytosis kit for flow cytometry (Thermofisher) for 30 min, followed by FACS analysis, as described previously (26). This determined the fraction of neutrophils with internalized bacteria (phagocytic rate) and average load of ingested bacteria per cell (MFI of pHrodo^+^ neutrophil population, phagocytic capacity).

Leucocyte gene expression was evaluated by quantitative polymerase chain reaction (qPCR) of whole blood mRNA from 39 blood samples on day 8 (F-GR = 6, F-CON = 33, P-GR = 9, P-CON = 30) and 37 on day 19 (F-GR = 6, F-CON = 31, P-GR = 9, P-CON = 28). Briefly, fresh blood was fixed by addition of a lysis binding solution (Thermofisher) to fresh whole blood and frozen (−80°C) for later processing, as previously described (27). Later, RNA was extracted (MagMAX 96 Blood RNA Isolation Kit, Thermofisher) and converted to cDNA according to the manufacturer's instructions (High capacity cDNA reverse transcription kit, Applied Biosystems, USA). We failed to extract RNA from one sample on day 8 (in F-CON and P-CON groups) and excluded this from analysis. Using a LightCycler 480 system (Roche, Switzerland) with a commercial qPCR kit (QuantiTect SYBR Green PCR Kit, Qiagen, Netherlands), gene expressions were determined for tumor necrosis factor alpha (*TNFA*), interleukin-4 (*IL4*), interleukin-6 (*IL6*), interleukin-10 (*IL10*), Interferon gamma (*IFNG*), GATA binding protein-3 (*GATA3*), T-box transcription factor TBX21 (*TBET*), toll-like receptor 2 (*TLR2*), toll-like receptor 4 (*TLR4*), and using hypoxanthine phosphoribosyltransferase 1 (*HPRT1*) as a housekeeping gene. Primers were designed using the *Genes* database and Primer-BLAST software (both National Center for Biotechnology Information, USA). All primers used are listed in Table 3. Samples were run in duplicates with expected 2.5% of variation of Cq values. For all samples, *HPRT1* was quantified within this variation limit. For the remaining genes, data were excluded if the variation of Cq values were higher than 2.5%. For information on the number of censored samples, please see Supplementary Table 1. Results were presented as fold changes, relative to expression levels of *HPRT1*. Ratios of *TBET*/*GATA3, IL2*/*IL4, IFNG*/*IL4*, and *TNFA*/*IL6* were used for indication of type 1 or type 2 T cell polarization (Th1 and Th2, respectively). The ratio of TNF-α to IL-6 is especially relevant in newborn infants (28, 29).

**Table 3:** Primers used for qPCR analyses.

**Gene**	**Forward primer**	**Reverse primer**
*TNFA*	ATTCAGGGATGTGTGGCCTG	CCAGATGTCCCAGGTTGCAT
*IL2*	AAGCTCTGGAGGGAGTGCTA	CAACAGCAGTTACTGTCTCATCA
*IL4*	GTACCAGCAACTTCGTCCAC	CCTTCTCCGTCGTGTTCTCT
*IL6*	TGCCACCTCAGACAAAATGC	AGGTTCAGGTTGTTTTCTGCC
*IL10*	GTCCGACTCAACGAAGAAGG	GCCAGGAAGATCAGGCAATA
*IFNG*	AGCTTTGCGTGACTTTGTGT	ATGCTCCTTTGAATGGCCTG
*TBET*	CTGAGAGTCGCGCTCAACAA	ACCCGGCCACAGTAAATGAC
*GATA3*	ACCCCTTATTAAGCCCAAGC	TCCAGAGAGTCGTCGTTGTG
*HPRT1*	TATGGACAGGACTGAACGGC	ACACAGAGGGCTACGATGTG

Plasma levels of interleukins 2, 6, and 10 (IL-2, IL-6, and IL-10), C reactive protein (CRP), cortisol and tumor necrosis factor alpha (TNF-α) were determined using ELISA (porcine specific duoset kits, R&D Systems). Samples below the detection were assigned an arbitrary value of half of the detection limit for statistical analyses.

### Statistics

Statistical analyses were performed using Stata 14.2 (StataCorp, Texas, USA). All data were evaluated for each time point separately, using linear regression models, with pig group (F-GR vs. F-CON or P-GR vs. P-CON) and litter as fixed effects. Data were logarithmically transformed, if necessary. Data without normal distribution were evaluated by Kruskal–Wallis' test. Mortality and group distribution were compared by Chi^2^ test. Means and corresponding standard error of means (SEMs) were reported. *P*-values <0.05 were considered statistically significant, and *p-*values < 0.1 was reported as a tendency to a difference.

## Results

### Effects of Fetal Growth Restriction on Clinical Variables and Organ Development

**Figure 1:**
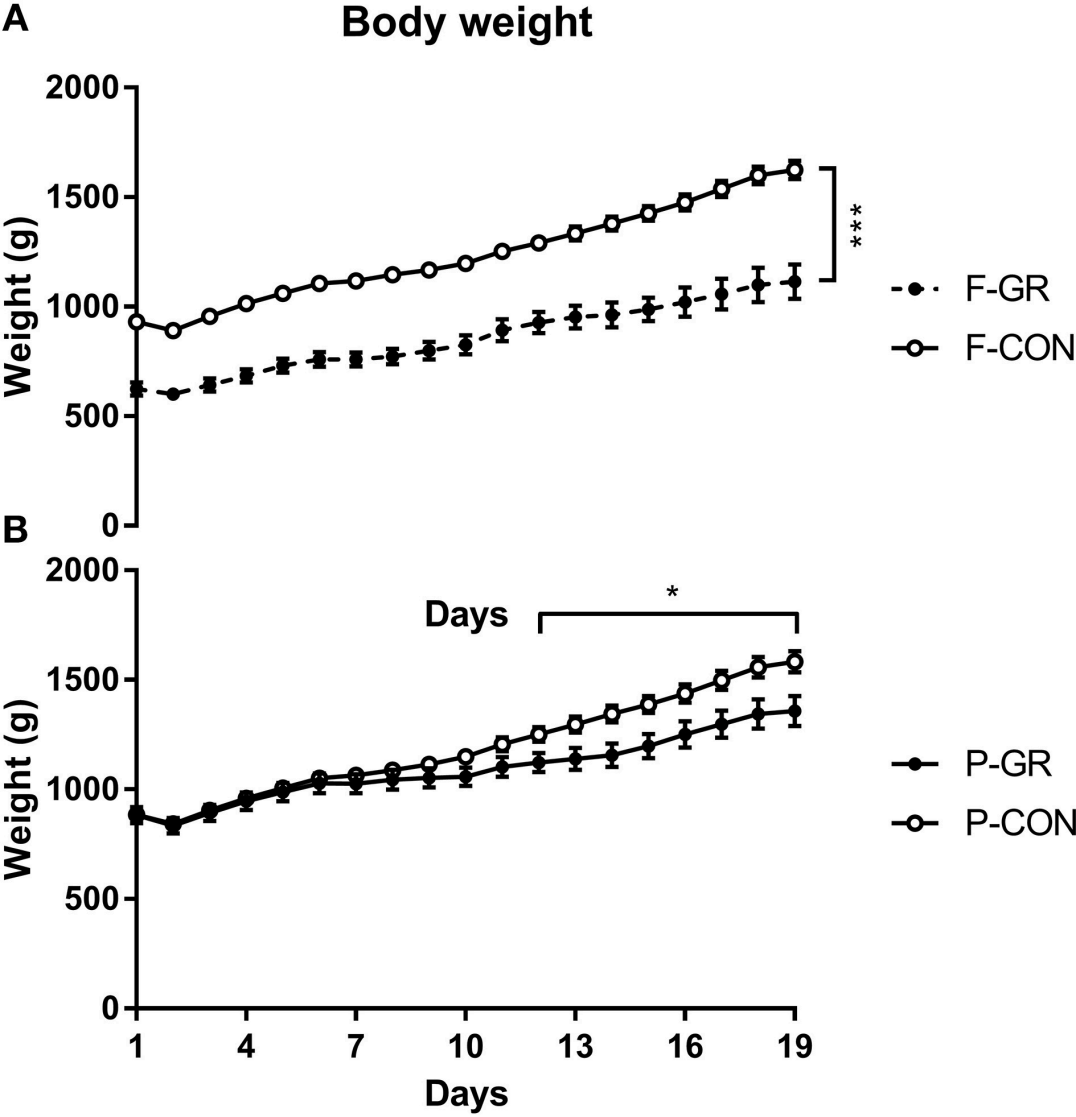
Body weight across the study period in fetal growth restricted preterm pigs and controls (F-GR, *n* = 27; F-CON, *n* = 92, **A**) and postnatally growth restricted preterm pigs and controls (P-GR, *n* = 24; P-CON, *n* = 85, **B**). Values are means with corresponding standard error of the mean. *p < 0.05, ***p < 0.001.

**Figure 2:**
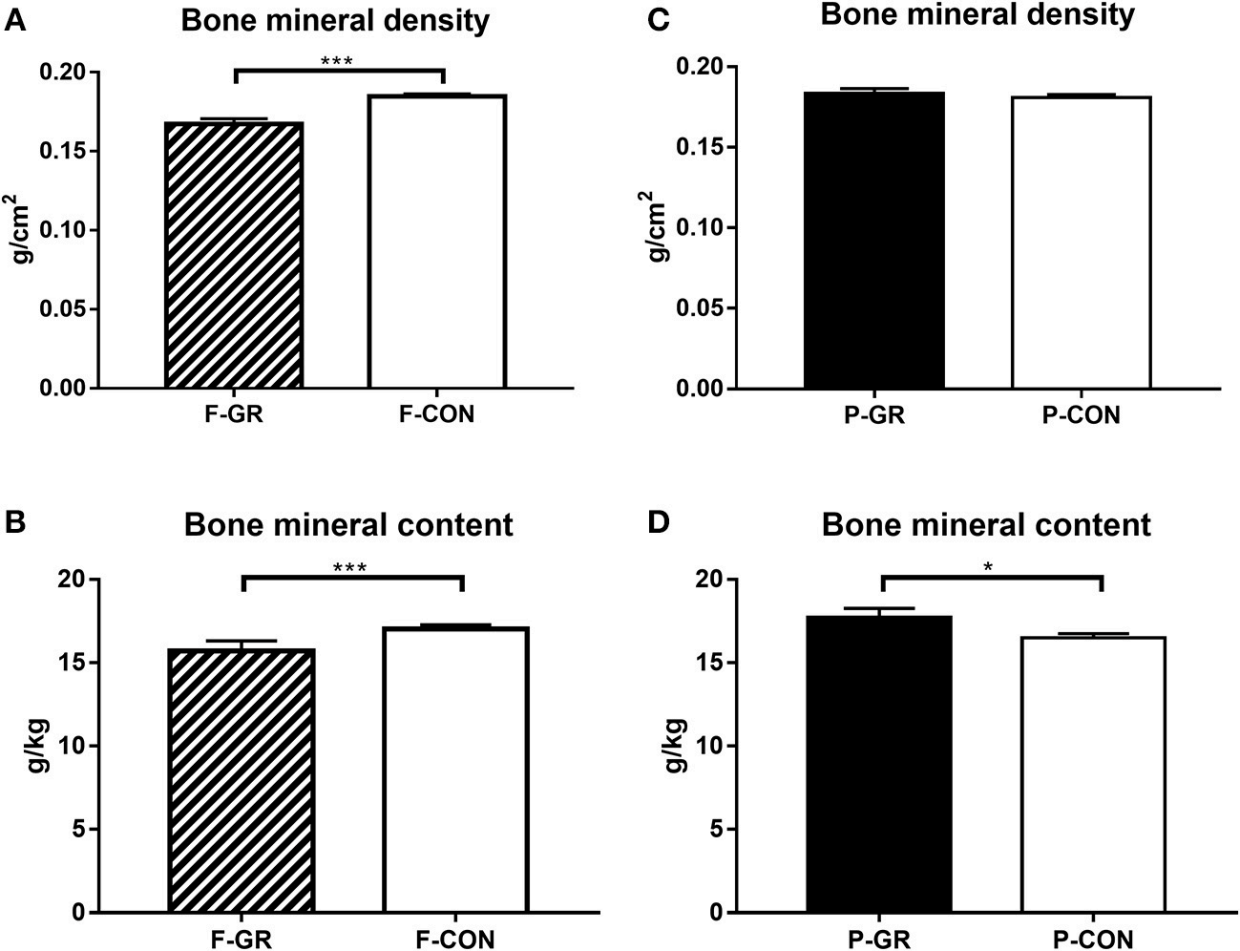
Bone mineral density and bone mineral content (relative to body weight) at euthanasia in fetal growth restricted preterm pigs and controls (F-GR, *n* = 16; F-CON, *n* = 70, **A,B**) and in postnatally growth restricted preterm pigs and controls (P-GR, *n* = 20; P-CON, *n* = 66, **C,D**). Values are means with corresponding standard error of the mean. *p < 0.05, ***p < 0.001.

Birth weight of F-GR piglets were 648 ± 26 vs. 930 ± 17 g in F-CON pigs (*p* < 0.001), but the subsequent relative daily weight gain until day 14 did not differ between the groups (27 ± 1 vs. 29 ± 1 g/kg/day). Body weight differed between F-GR and F-CON piglets for all time points (Figure 1A). Days with diarrhea (mean 6 days in both groups) and CRP levels (3.2 ± 1.1 vs. 7.2 ± 2.0 μg/mL on day 19 for F-GR and F-CON) did not differ, but mortality was higher in the F-GR group (7 of 27 before day 14 vs. 6 of 95 for F-CON pigs, *p* < 0.01). On day 19, surviving F-GR pigs had lower body weight (1,113 ± 80 vs. 1,624 ± 41 g, *p* < 0.001), while relative adrenal gland and kidney weight was higher (Table 4, *p* < 0.001 and *p* = 0.07, respectively) than in F-CON pigs. Plasma cortisol levels at day 19 did not differ between F-GR and F-CON pigs (23 ± 4 vs. 33 ± 8 ng/mL). For body composition, F-GR pigs had lower bone mineral density and relative bone mineral content than F-CON pigs (Figures 2A,B, both *p* < 0.001). Lean body mass (95.3 ± 0.3 vs. 95.4 ± 0.2%) and fat mass (3.9 ± 0.3 vs. 3.7 ± 0.2%) were similar.

**Table 4:** Relative weight (g per kg body weight) of internal organs on day 19.

	**F-GR, *n* = 19**	**F-CON, *n* = 86**	**P-GR, *n* = 24**	**P-CON, *n* = 81**
Heart	6.8 ± 0.4	6.5 ± 0.1	6.6 ± 0.2	6.5 ± 0.1
Lung	19.1 ± 1.5	18.6 ± 0.6	20.4 ± 1.6^(*)^	17.8 ± 0.6
Kidney	8.2 ± 0.4^(*)^	7.2 ± 0.1	7.9 ± 0.3^**^	7.1 ± 0.1
Adrenals	0.40 ± 0.02^***^	0.29 ± 0.01	0.31 ± 0.02	0.30 ± 0.01
Spleen	2.6 ± 0.3	2.8 ± 0.1	2.7 ± 0.3	2.8 ± 0.1
Liver	23.4 ± 1.3	25 ± 0.6	23.5 ± 1.3	24.5 ± 0.6
Stomach	7.7 ± 0.4	6.8 ± 0.1	7 ± 0.3	6.8 ± 0.1
Intestine	40.9 ± 1.8	41.7 ± 0.9	42.6 ± 1.9	42.0 ± 0.9
Colon	17.9 ± 1.8	20 ± 1.2	22.1 ± 2.4	19.4 ± 1.1

Data is presented as means ± SEM.

** p < 0.01,

*** p < 0.001,

(*) *p < 0.10 for F-GR vs. F-CON or P-GR vs. P-CON comparisons, respectively. Data shown for fetal growth restricted preterm pigs and controls (F-GR, F-CON) and postnatally growth restricted preterm pigs and controls (P-GR, P-CON). SEM, Standard error of the mean*.

### Effects of Fetal Growth Restriction on Blood Hematology, Biochemistry, and Immune Development

**Figure 3:**
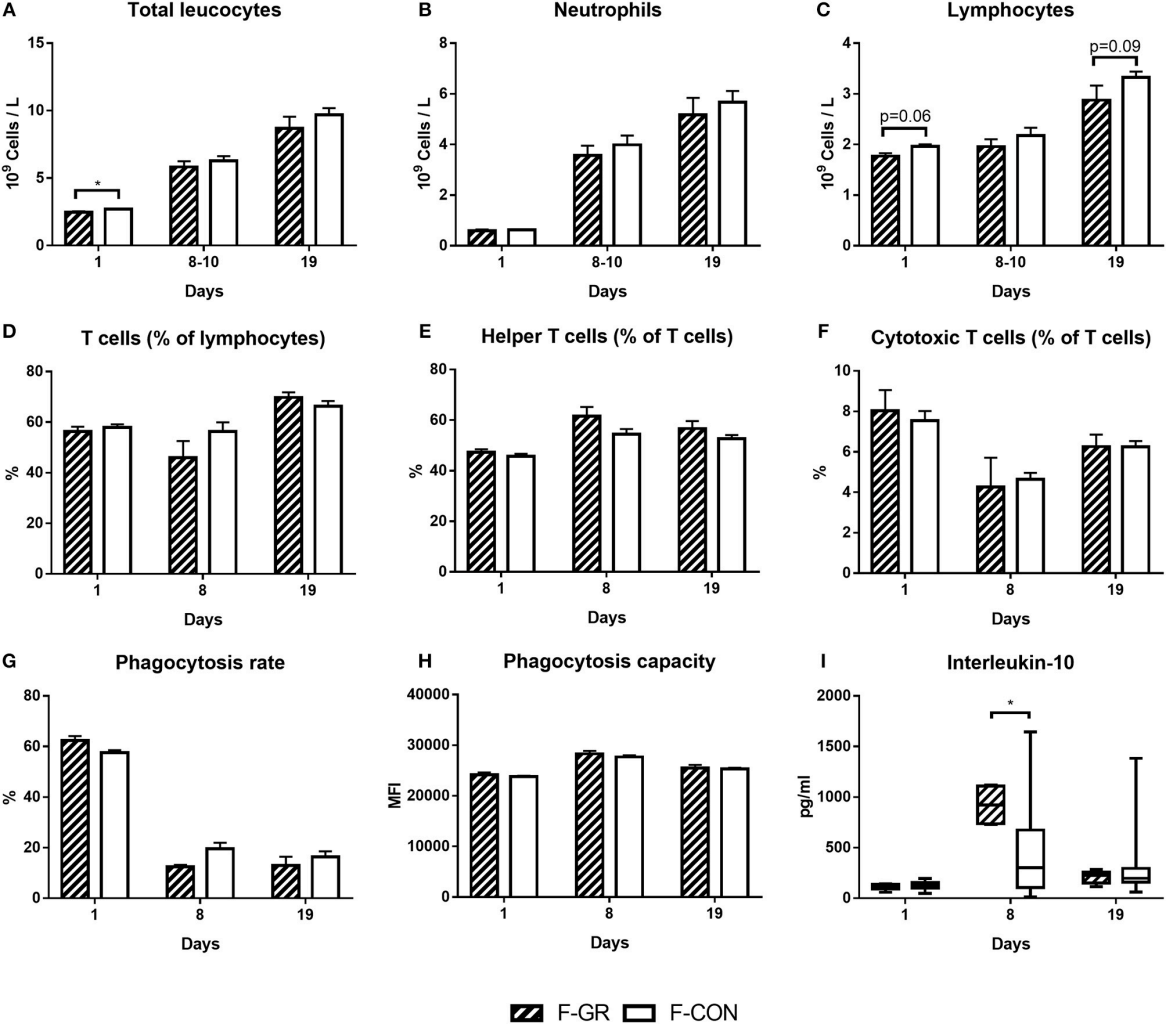
Development of systemic immune parameters in fetal growth restricted preterm pigs and controls (F-GR, *n* = 19–24 for **A–C** and *n* = 6–10 for **D–I**; F-CON, *n* = 71–89 for **A–C** and *n* = 34–37 for **D–I**), day 1 (cord blood) to day 19. **(A–C)** leucocyte cell counts, **(D–F)** fractions of T cell subsets, **(G,H)** neutrophil phagocytosis function, I: interleukin-10 levels in plasma (two samples were below detection limit on day 8, both in F-CON group). Values in **(A–H)** are presented as means with corresponding standard error of the mean. Values in I are presented as range plots with corresponding means, all analyzed by Kruskal–Wallis' test. *p < 0.05.

F-GR pigs had lower total blood leucocyte counts (Figure 3A, *p* < 0.05) at birth, mainly driven by lower lymphocyte counts (Figure 3C, *p* = 0.06). Cytotoxic T cell MFI tended to be higher in F-GR animals (847 ± 120 vs. 768 ± 133, *p* = 0.06). No other blood immune cell counts, T cell fractions, T cell MFI, plasma cytokine levels or markers for blood neutrophil phagocytic function differed at birth (Figures 3B,D–H). By day 8, only plasma IL-10 levels were higher in F-GR vs. F-CON pigs (Figure 3I, *p* < 0.05), whereas all other leucocyte counts (Figures 3A–C), T cell subsets (Figures 3D–F), neutrophil phagocytic function (Figures 3G,H) and plasma cytokine levels and T cell MFI (data not shown) were similar between the two groups, both at day 8–10 and day 19. Ratios of TNFα/IL-10 did not differ at any time points.

**Figure 4:**
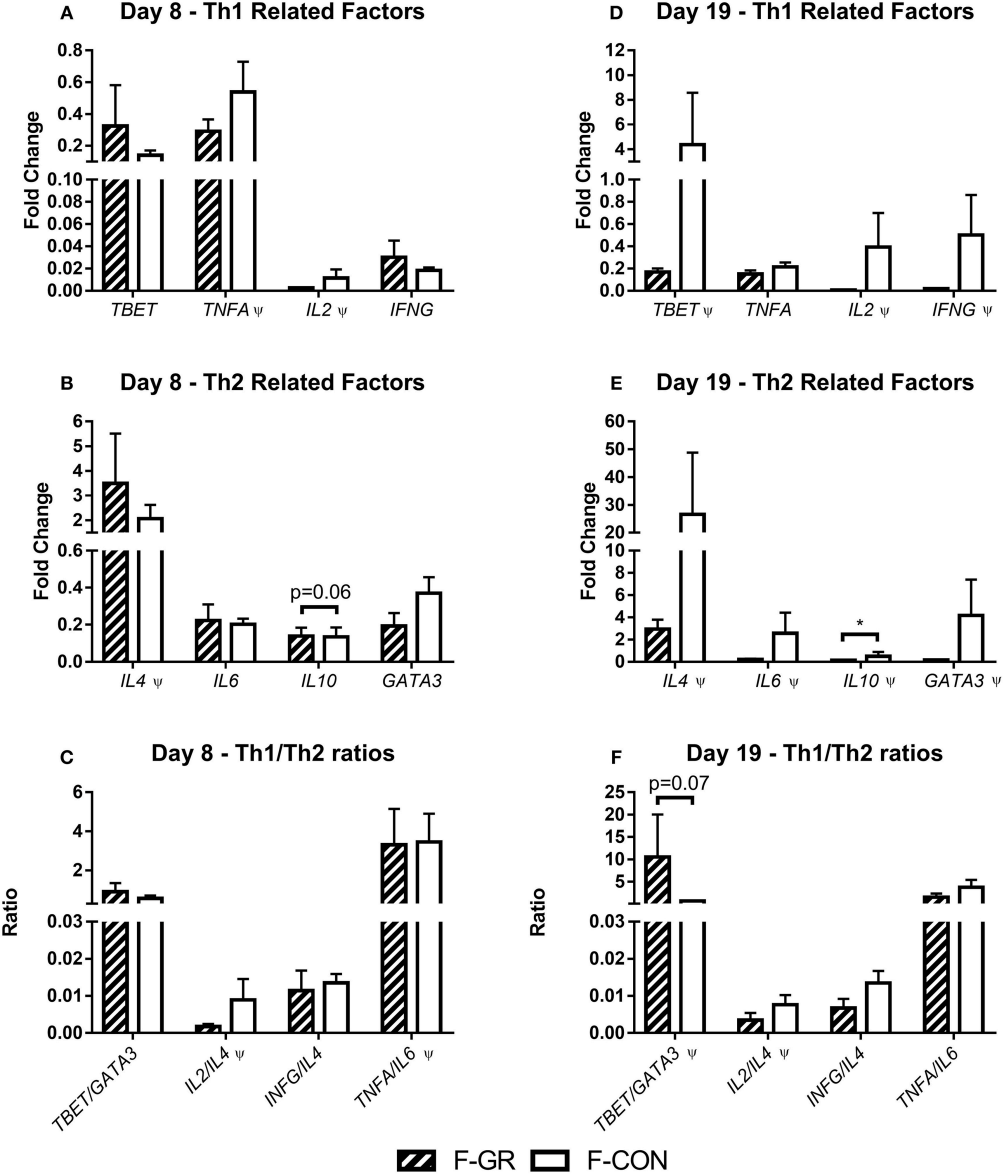
Leucocyte gene expression in fetal growth restricted preterm pigs and controls on day 8 (**A–C**, F-GR, *n* = 5–6; F-CON, *n* = 26–32) and 19 (**D–F**, F-GR, *n* = 5–6; F-CON, *n* = 27–31). **(A,D)** type 1 helper T cell related genes, **(B,E)** type 2 helper T cell related genes, **(C,F)** ratios of type 1 and type 2 helper T cell related genes. Results presented as mean fold changes in relation to housekeeping gene with corresponding standard error of the mean **(A,B,D,E)** or as ratios between fold changes **(C,F)**. *p < 0.05, Ψ, analyzed by Kruskal–Wallis' test.

For all tested leucocyte gene expressions, no differences at day 8 were observed (Figures 4A,C), apart from a tendency to higher *IL10* expression in F-GR, relative to F-CON pigs (Figure 4B, *p* = 0.06). By day 19, *IL10* expression was lower (Figure 4E, *p* < 0.05) and there was a tendency to an increased *TBET*/*GATA3* ratio in F-GR pigs (Figure 4F, *p* = 0.07). Likewise, there was a tendency toward a lower *TNFA/IL10* ratio in F-GR pigs at day 19 (2.9 ± 1.0 vs. 3.8 ± 0.9, *p* = 0.09)

For non-immunological parameters, F-GR pigs showed higher values of mean platelet volume than F-CON pigs at birth and day 8–10 (MPV, Table 5, both *p* < 0.05) but no serum biochemical values at day 19 showed any differences between F-GR and F-CON pigs.

**Table 5:** Hematological parameters at day 1, 8–10, and 19.

	**Day**	**F-GR *n* = 19–24**	**F-CON *n* = 71–89**	**P-GR *n* = 18–23**	**P-CON *n* = 67–80**
Erythrocytes (10^12^ cells/L)	1^†^	4.1 ± 0.1	4.0 ± 0.0	4.0 ± 0.1	4.1 ± 0.0
	8–10	4.1 ± 0.2	4.0 ± 0.1	4.0 ± 0.2	4.1 ± 0.1
	19	4.5 ± 0.2	4.5 ± 0.1	4.5 ± 0.1	4.5 ± 0.1
Hemoglobin (mmol/L)	1^†^	5.5 ± 0.1	5.4 ± 0.0	5.4 ± 0.1	5.5 ± 0.1
	8–10	4.8 ± 0.3	4.7 ± 0.1	4.8 ± 0.2	4.8 ± 0.1
	19	4.7 ± 0.2	4.7 ± 0.1	4.6 ± 0.2	4.8 ± 0.1
Hematocrit (%)	1^†^	29.9 ± 0.4	29.5 ± 0.3	29.2 ± 0.4	29.6 ± 0.3
	8–10	25.9 ± 1.5	25.1 ± 0.5	25.5 ± 1.1	25.5 ± 0.5
	19	25.6 ± 1	26.0 ± 0.5	26.0 ± 1.0	25.9 ± 0.5
MCHC (mmol/L)	1^†^	18.4 ± 0.3	18.2 ± 0.2	17.7 ± 0.8	18.5 ± 0.1
	8–10	18.7 ± 0.4	18.7 ± 0.1	18.7 ± 0.3	18.8 ± 0.1
	19	18.6 ± 0.4	18.3 ± 0.3	17.9 ± 0.8	18.5 ± 0.2
MCV (fL)	1^†^	72.3 ± 1.0	73.5 ± 0.7	72.5 ± 1.1	72.9 ± 0.7
	8–10	62.4 ± 1.4	63.2 ± 0.7	63.6 ± 1.2	62.9 ± 0.7
	19	57.5 ± 1.2	58.2 ± 0.6	57.5 ± 1.0	58.2 ± 0.7
Platelets (10^9^ cells/L)	1^†^	187 ± 23	210 ± 11	191 ± 23	201 ± 11
	8–10	343 ± 41	359 ± 22	456 ± 56^(*)^	341 ± 18
	19	432 ± 60	487 ± 28	528 ± 65	460 ± 27
MPV (fL)	1^†^	10.5 ± 0.3^*^	10.1 ± 0.1	10.2 ± 0.2	10.1 ± 0.1
	8–10	13.1 ± 1.3^**^	10.6 ± 0.4	11.9 ± 1.2	10.5 ± 0.3
	19	9.7 ± 0.6	9.6 ± 0.3	9.6 ± 0.6	9.6 ± 0.3
MPC (g/L)	1^†^	220 ± 2	220 ± 1	219 ± 2	220 ± 1
	8–10	224 ± 5	224 ± 2	228 ± 5	223 ± 2
	19	228 ± 4	231 ± 2	230 ± 3	230 ± 2

Data is presented as means ± SEM.

† *Cord blood sample*;

(*) *p < 0.1*,

* p < 0.05, and

** *p < 0.01 for F-GR vs. F-CON or P-GR vs. P-CON comparisons, respectively. Data shown for fetal growth restricted preterm pigs and controls (F-GR; F-CON) and postnatally growth restricted preterm pigs and controls (P-GR, P-CON). MCHC, Mean cellular hemoglobin concentration; MCV, Mean cellular volume; MPV, Mean platelet volume; MPC, Mean platelet component; SEM, Standard error of the mean*.

### Effects of Postnatal Growth Restriction on Clinical Variables and Organ Development

Birth weight did not differ between P-GR and P-CON pigs (883 ± 37 vs. 883 ± 21 g). P-GR pigs had reduced postnatal relative weight gain (20 ± 1 vs. 31 ± 1 g/kg/day in P-CON pigs, *p* < 0.001), and lower body weight at day 19 (1,357 ± 68 vs. 1,583 ± 48 g in P-CON pigs, *p* < 0.05). Body weight started to differ after day 12 (Figure 1B). Days with diarrhea (6–7 days in both groups) and CRP levels (12.1 ± 4.7 vs. 5.2 ± 1.7 μg/mL) were similar between P-GR and P-CON pigs. Among the 20 surviving F-GR pigs, six of them were categorized as P-GR, thus the incidence of P-GR did not differ significantly between F-GR and F-CON pigs (*p* > 0.1). Relative kidney weight was increased in P-GR animals (Table 4, *p* < 0.05) with a tendency toward increased relative lung weight (Table 4, *p* = 0.06). For body composition, bone mineral content was increased in P-GR pigs (Figure 2D, *p* < 0.05), while values for bone mineral density (Figure 2C), lean mass (95.7 ± 0.4 vs. 95.3 ± 0.2%) and fat mass (3.5 ± 0.3 vs. 3.8 ± 0.2%) were similar.

### Effects of Postnatal Growth Restriction on Blood Hematology, Biochemistry, and Immune Development

**Figure 5:**
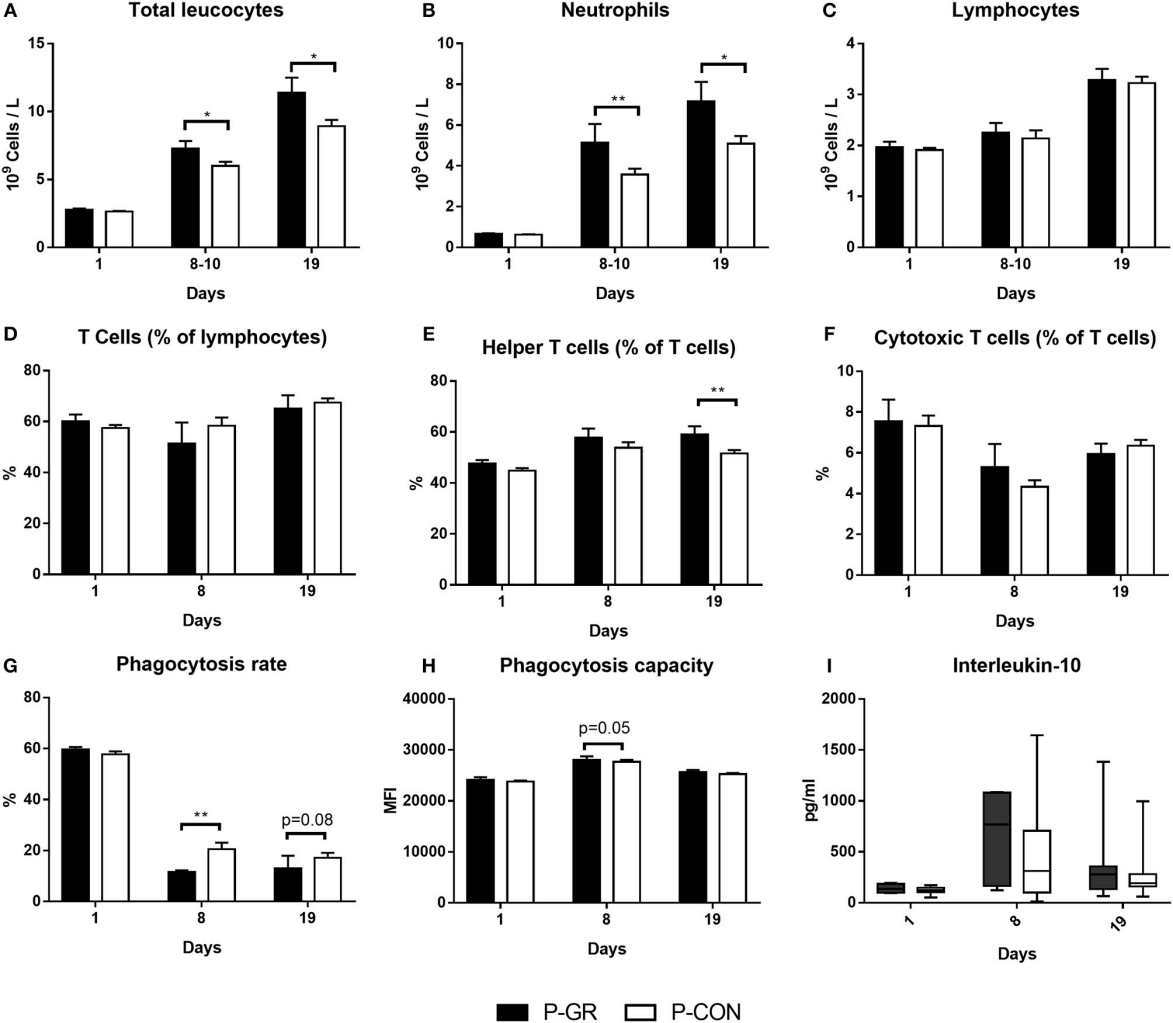
Development of systemic immune parameters in postnatally growth restricted preterm pigs and controls (P-GR, *n* = 18–23 for A–C and *n* = 9–10 for **D–I**; P-CON, *n* = 67–80 for **A–C** and *n* = 29–34 for **D–I**) from day 1 (cord blood) to day 19. **(A–C)** leucocyte cell counts, **(D–F)** fractions of T cell subsets, **(G,H)** neutrophil phagocytosis function, I: interleukin-10 levels in plasma (two samples were below detection limit on day 8, both in P-CON group). In **(A–H)**, values are presented as means with corresponding standard error of the mean. In **(I)**, values are presented as range plots with corresponding means, all analyzed by Kruskal–Wallis' test. *p < 0.05, **p < 0.01.

At birth, no differences in blood immune cell parameters, neutrophil phagocytic function, T cell fractions, T cell MFI or plasma cytokine levels were found between P-GR and P-CON pigs (Figures 5A–I). By day 8–10, there was higher total blood leucocyte counts in the P-GR group (Figure 5A, *p* < 0.05), mainly driven by increased neutrophil counts (Figure 5B, *p* < 0.01). In addition, the neutrophil phagocytic rate (Figure 5G, *p* < 0.01) was reduced, with a tendency to improved phagocytic capacity (Figure 5H, *p* = 0.05) in P-GR pigs. At day 19, total leucocyte and neutrophil counts remained higher in P-GR pigs (Figures 5A,B, both *p* < 0.05), with a tendency to lower neutrophil phagocytic rate (Figure 5G, *p* = 0.08). In addition, monocyte (0.50 ± 0.09 vs. 0.34 ± 0.03 × 10^9^ cells/L, *p* < 0.05) and eosinophil (0.16 ± 0.03 vs. 0.09 ± 0.01 × 10^9^ cells/L, *p* < 0.05) counts, as well as the fraction of helper T cells (Figure 5E, *p* < 0.01), were increased in P-GR animals. Plasma levels of IL-10 (Figure 5I) or other cytokines, as well as T cell MFI (data not shown) did not differ at day 8 or 19 between P-GR and P-CON pigs. Likewise, ratios of TNFα/IL-10 did not vary at any time point. The P-GR animals tended to have lower platelet counts (Table 5, *p* = 0.05) at day 8–10, relative to P-CON pigs. Other than lower alkaline phosphatase levels in P-GR pigs at day 19, no biochemical variables or plasma cortisol level differed between groups (Table 6, *p* < 0.05).

**Table 6:** Blood biochemistry at day 19.

	**F-GR *n* = 22**	**F-CON *n* = 81**	**P-GR *n* = 22**	**P-CON *n* = 77**
Albumin (g/L)	16.6 ± 0.9	17.6 ± 0.4	17.3 ± 1	17.4 ± 0.4
Total protein (g/L)	27.9 ± 1.2	29.5 ± 0.7	29.4 ± 1.7	29.1 ± 0.6
BUN (mmol/L)^Ψ^	7.4 ± 0.9	6.8 ± 0.5	8.1 ± 1.0	6.5 ± 0.5
ALAT (U/L)	34 ± 3	32 ± 1	33 ± 2	32 ± 1
AST (U/L)^Ψ^	50 ± 8	35 ± 2	40 ± 5	37 ± 2
Alkaline phosphatase (U/L)	1,085 ± 122	1,080 ± 63	956 ± 116^*^	1,117 ± 63
Bilirubin (μmol/L)^Ψ^	1.3 ± 0.2	1.6 ± 0.2	1.5 ± 0.3	1.6 ± 0.2
GGT (U/L)^Ψ^	20.1 ± 1.6	22.4 ± 1	21.9 ± 2.3	22.0 ± 0.9
Cholesterol (mmol/L)^Ψ^	2.5 ± 0.1	2.7 ± 0.1	2.6 ± 0.1	2.6 ± 0.1
Creatine (mmol/L)^Ψ^	62.5 ± 10.3	53.2 ± 1.4	65.1 ± 9.0	52.1 ± 1.4
Creatine kinase (mmol/L)^Ψ^	375 ± 145	213 ± 25	223 ± 48	250 ± 43
Glucose (mmol/L)^Ψ^	4.6 ± 0.5	5.2 ± 0.2	4.8 ± 0.4	5.1 ± 0.2
Lactate (mmol/L)^Ψ^	4.6 ± 1.0	4.9 ± 0.4	5.1 ± 1.0	4.8 ± 0.4
Sodium (mmol/L)^Ψ^	140 ± 2	143 ± 2	142 ± 4	143 ± 2
Potassium (mmol/L)	4.4 ± 0.3	4.7 ± 0.1	4.6 ± 0.3	4.6 ± 0.1
Phosphate (mmol/L)	2.3 ± 0.1	2.5 ± 0.1	2.4 ± 0.1	2.5 ± 0.1
Calcium (mmol/L)	2.5 ± 0.1	2.6 ± 0.0	2.5 ± 0.1	2.6 ± 0.0
Magnesium (mmol/L)	0.9 ± 0.0	0.9 ± 0.0	1.0 ± 0.1	0.9 ± 0.0
Iron (mmol/L)^Ψ^	10.2 ± 1.3	9.1 ± 0.7	9.4 ± 1.2	9.3 ± 0.7

Data is presented as means ± SEM.

* p < 0.05 for P-GR vs. P-CON comparison.

Ψ *comparison done by Kruskal–Wallis' test. Data shown for fetal growth restricted preterm pigs and controls (F-GR, F-CON) and postnatally growth restricted preterm pigs and controls (P-GR, P-CON). ALAT, alanine aminotransferase; AST, aspartate aminotransferase; BUN, blood urea nitrogen; GGT, gamma glutaminyl transferase; SEM, standard error of the mean*.

**Figure 6:**
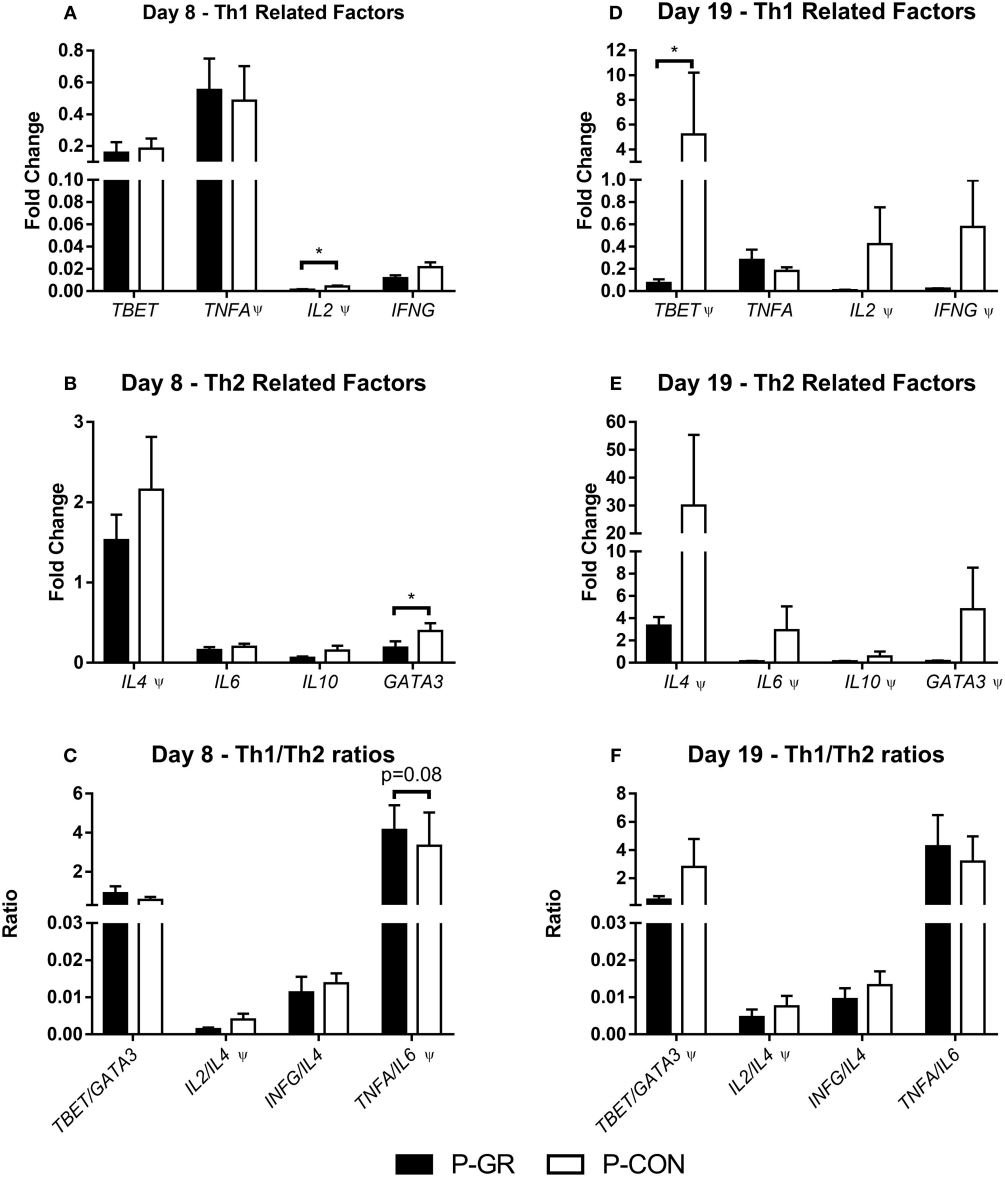
Leucocyte gene expression in postnatally growth restricted preterm pigs and controls, on day 8 (**A–C**, P-GR, *n* = 8–9; P-CON, *n* = 24–29) and 19 (**D–F**, P-GR, *n* = 7–9; P-CON, *n* = 25–28). **(A,D)** type 1 helper T cell related genes, **(B,E)** type 2 helper T cell-related genes, **(C,F)** ratios of type 1 and type 2 helper T cell related genes. Results presented as mean fold changes in relation to housekeeping gene with corresponding standard error of the mean **(A,B,D,E)**, or as ratios between fold changes **(C,F)**. *p < 0.05, Ψ, analyzed by Kruskal–Wallis' test.

Leucocyte gene expression at day 8 showed reduced expression of *IL2* (Figure 6A, *p* < 0.05) and *GATA3* (Figure 6B, *p* < 0.05), together with a tendency toward an increased ratio of *TNFA* to *IL6* (Figure 6C, *p* = 0.08) in P-GR pigs. By day 19 several genes were expressed at numerically lower levels in P-GR animals, but only *TBET* (Figure 6D, *p* < 0.05) expression was significantly down regulated in the P-GR piglets. No differences were observed for Th2 related factors (Figure 6E). Ratios of TNFA/IL10 did not differ at any time point between P-GR and P-CON (data not shown).

## Discussion

Preterm infants, especially those born extremely preterm (<28 weeks gestation), show a high sensitivity to infections in the postnatal period. A large proportion of these are born growth restricted and show growth deficits after birth due to multiple maternal, fetal, and postnatal factors (4, 30). It is important to know if low growth rates are associated with impaired immune development because this would call for special clinical interventions to avoid infections. Using preterm pigs as a model for preterm infants, we show that a moderate growth restriction at birth, excluding extremely low birth weight preterm pigs, was associated with increased adrenal gland weight, reduced bone mineralization, and a transient change in circulating IL-10 levels. Conversely, slow postnatal growth rates were associated with modest increases in bone mineralization, blood neutrophil, monocyte, and eosinophil counts, and a transiently higher helper T cell fraction. Taken together, surprisingly few parameters differed between F-GR/P-GR and control littermate pigs, suggesting that moderate growth restriction before or after preterm birth is not associated with major developmental defects in the systemic immune system. In addition, postnatal growth restriction was in fact associated with an increase in several immune cell types, possibly indicating accelerated immune cell maturation. However, it remains elusive whether those changes related to growth restriction may lead to altered immune competence against infectious challenges. Further studies with *in vivo* infection challenges are required to allow conclusion about mechanisms possibly causing increased risk of infection in growth restricted infants as previously documented (1, 8–10). Nevertheless, our data indicate that with appropriate clinical care and nutrition, moderately growth restricted preterm infants may show great capacity for short term systemic immune adaptation, although long term effects are unknown.

Following fetal growth restriction, we found a lower total leucocyte and lymphocyte counts at birth in F-GR pigs, but with no differences in T cell subsets or plasma cytokine levels. By day 8, plasma levels and whole blood gene expressions of IL-10 were elevated in F-GR pigs. By day 19 however, the expression of *IL10* was reduced in the F-GR animals and the *TBET/GATA3* ratio tended to be increased, possibly indicating higher Th1 activity. Overall, the leucocyte gene expressions on day 19 showed lower expressions of most genes. IL-10 has been considered as an anti inflammatory cytokine produced by Th2 and regulatory T cells (31), but is also expressed by many other adaptive and innate immune cells (32). However, IL-10 production in innate cells is driven primarily by microbial activation of macrophages and dendritic cells (32, 33). The lack of clinical, CRP or immune cell responses suggest that the IL-10 changes observed may be derived from regulatory T or Th2 cells. Any systemic immune suppressive state shortly after preterm birth may disappear after 19 days. Such effects may relate to increased adrenocortical activity after fetal growth restriction, as indicated by increased relative adrenal gland weight in F-GR pigs, despite the unaffected basal cortisol levels detected at day 19. Apparently, the catabolic effects of increased adrenocortical activity in F-GR piglets did not induce notable postnatal growth restriction as F-GR pigs were not subject to more frequent postnatal growth restriction than F-CON pigs. Likewise, biochemical indices at day 19 were unaffected by the slow fetal growth rate.

During postnatal growth restriction, bone growth may be prioritized, supported by the lower levels of alkaline phosphatase, possibly indicating reduced osteoblastic activity (34). Likewise, the kidneys appeared to be a prioritized organ in postnatal growth restricted animals, as indicated by elevated relative weight on day 19. More importantly, postnatal growth restriction was associated with changes in immune cell populations. On day 8–10 P-GR animals had higher neutrophil counts, but lower fractions of neutrophils with phagocytic capacity, suggesting increased recruitment of immature neutrophils (35, 36). Neutrophils mature in the bone marrow over a period of 30 days and are under normal homoeostatic circumstances kept in the bone marrow as a reserve and slowly released to the circulation, a process regulated by granulocyte colony stimulation factor (37). During immunological reactions, these mature neutrophils can be recruited to the circulation under the influence of other chemotactic agents (38). A higher level of immature neutrophils in P-GR pigs could indicate a smaller reservoir of mature neutrophils or an accelerated granulopoiesis. Apart from neutrophils, monocytes, basophils, eosinophil counts as well as helper T cell fractions were all elevated in P-GR pigs at day 19. However, plasma cytokine levels and leucocyte gene expression were not affected, or even tended to be down regulated at day 19 in P-GR vs. P-CON pigs. In the absence of clinical symptoms of infections, as well as few changes in organ weights and biochemical parameters, the slow growing preterm pigs managed to support and even increase proliferation of innate immune cells. Leucocyte gene expressions on day 8 showed reduced expression of *IL2*, a key Th1 cytokine, and *GATA3* a Th2 transcription factor. Thus, it seems that both Th1- and Th2-related factors were down regulated, while Th1/Th2 ratios were largely unaffected, apart from a tendency to an increased *TNFA/IL6* ratio. At day 19, a similar pattern of gene expressions was observed between P-GR and P-CON pigs. Similar to the trends in F-GR animals, most of the measured genes showed low mean expressions in the P-GR group. The similarity in leucocyte gene expression may be partly explained by the overlap of pigs included into both the P-CON and F-CON groups. On the other hand, the higher proportion of neutrophils observed in the P-GR group could also play a role in decreased the expression of the investigated lymphocyte related genes.

In conclusion, we observed limited effects of moderate fetal and postnatal growth restriction on organ growth, blood biochemistry and immune cell development during the first 3 weeks after birth in preterm pigs. We excluded preterm pigs with extreme growth restriction (<350 g, lowest 5%) due to mortality shortly after birth, hence our results may be translationally most relevant for preterm infants without serious complications and GR in the immediate neonatal period. Our experimental conditions may have influenced GR effects on some immune parameters, such as the use of antibiotics, which are known to influence immune system development (27). Further, the higher mortality of F-GR piglets during the first weeks may have differentially affected various immune parameters as recorded on day 19. Likewise, other than neutrophil phagocytic capacity, we did not perform *ex vivo* challenges to immune cells, so the impact of growth restriction on some immune cell functions (e.g., cytokine response) is unknown. Finally, given the overlap in groups and the wide variability in the leucocyte gene expressions, these data should be interpreted with caution. Nevertheless, the factors leading to moderate growth restriction (genetic or environmental influences before or after birth) did not prevent surviving preterm pigs from following a near normal immune developmental trajectory, compared to control animals in the early postnatal period. While extreme growth restriction (<10% growth percentile) and prematurity (<28 weeks gestation) may compromise immunity at many levels, our results indicate that neonates subjected to moderate growth restriction and immaturity have a remarkable capacity to adapt their systemic immune system during the first weeks after birth. The long term effects on cell population, immune function and susceptibility to infections remain to be elucidated in future studies.

## Data Availability Statement

The datasets generated for this study are available on request to the first and corresponding author.

## Ethics Statement

The animal study was reviewed and approved by Danish National Committee of Animal Experimentation.

## Author Contributions

OB, DN, and PS planned the research. TT supervised the experiments. OB and DN did data analysis and interpretation of results. OB wrote the manuscript. PS and DN had primary responsibility for the final content. All authors read and approved the final paper.

### Conflict of Interest

The authors declare that the research was conducted in the absence of any commercial or financial relationships that could be construed as a potential conflict of interest.

## Abbreviations

IUGR: Intrauterine growth restriction
EUGR: Extrauterine growth restriction
PN: Parenteral nutrition
F-GR: Fetal growth restriction (Group name)
F-CON: Fetal growth restriction control (Group name)
P-GR: Postnatal growth restriction (Group name)
P-CON: Postnatal growth restriction control (Group name)
MFI: Median fluorescent index
qPCR: Quantitative polymerase chain reaction
*TNFA*: Tumor necrosis factor alpha (gene)
IL4: Interleukin-4 (gene)
*IL6*: Interleukin-6 (gene)
*IL10*: Interleukin-10 (gene)
*IFNG*: Interferon gamma (gene)
*GATA*: GATA binding protein-3 (gene)
*TBET*: T-box transcription factor TBX21 (gene)
*TLR2*: Toll-like receptor 2 (gene)
*TLR4*: Toll-like receptor 4 (gene)
*HPRT1*: Hypoxanthine Phosphoribosyltransferase 1 (gene)
IL-2: Interleukin-2
IL-6: Interleukin-6
IL-10: Interleukin-10
TNF-α: Tumor necrosis factor alpha
CRP: C reactive protein
ELISA: Enzyme linked immune assay
MCHC: Mean cellular hemoglobin concentration
MCV: Mean cellular volume
MPV: Mean platelet volume
MPC: Mean platelet component, BUN, Blood urea nitrogen
ALAT: Alanine aminotransferase
AST: Aspartate aminotransferase
GGT: Gamma glutaminyl transferase.

## References

[1] Stoll BJ Hansen N Fanaroff AA Wright LL Carlo WA Ehrenkranz RA. Late-onset sepsis in very low birth weight neonates: the experience of the NICHD Neonatal Research Network. Pediatrics. (2002) 110:285–91. 10.1542/peds.110.2.285 12165580

[2] Strunk T Currie A Richmond P Simmer K Burgner D. Innate immunity in human newborn infants: prematurity means more than immaturity. J Matern Neonatal Med. (2011) 24:25–31. 10.3109/14767058.2010.482605 20569168

[3] Tröger B Müller T Faust K Bendiks M Bohlmann MK Thonnissen S. Intrauterine growth restriction and the innate immune system in preterm infants of ≤32 weeks gestation. Neonatology. (2013) 103:199–204. 10.1159/000343260 23295537

[4] Gilbert WM Danielsen B. Pregnancy outcomes associated with intrauterine growth restriction. Am J Obstet Gynecol. (2003) 188:1596–601. 10.1067/mob.2003.384 12824998

[5] Wirbelauer J Thomas W Rieger L Speer CP. Intrauterine growth retardation in preterm infants ≤32 weeks of gestation is associated with low white blood cell counts. Am J Perinatol. (2010) 27:819–24. 10.1055/s-0030-1254547 20480452

[6] Ferguson AC. Prolonged impairment of cellular immunity in children with intrauterine growth retardation. J Pediatr. (1978) 93:52–6. 10.1016/S0022-3476(78)80599-X 77323

[7] Harbeson D Francis F Bao W Amenyogbe NA Kollmann TR. Energy demands of early life drive a disease tolerant phenotype and dictate outcome in neonatal bacterial sepsis. Front Immunol. (2018) 9:1918. 10.3389/fimmu.2018.01918 30190719 PMC6115499

[8] Tröger B Göpel W Faust K Müller T Jorch G Felderhoff-Müser U. Risk for late-onset blood-culture proven sepsis in very-low-birth weight infants born small for gestational age: a large multicenter study from the German Neonatal Network. Pediatr Infect Dis J. (2014) 33:238–43. 10.1097/INF.0000000000000031 24030351

[9] Simchen MJ Beiner ME Strauss-Liviathan N Dulitzky M Kuint J Mashiach S. Neonatal outcome in growth-restricted versus appropriately grown preterm infants. Am J Perinatol. (2000) 17:187–92. 10.1055/s-2000-9423 11041440

[10] Weisz B Hogen L Yinon Y Gindes L Shrim A Simchen M. Perinatal outcome of monochorionic twins with selective IUGR compared with uncomplicated monochorionic twins. Twin Res Hum Genet. (2011) 14:457–62. 10.1375/twin.14.5.457 21962139

[11] Cromi A Ghezzi F Raffaelli R Bergamini V Siesto G Bolis P. Ultrasonographic measurement of thymus size in IUGR fetuses: a marker of the fetal immunoendocrine response to malnutrition. Ultrasound Obstet Gynecol. (2009) 33:421–6. 10.1002/uog.6320 19306477

[12] Ekin A Gezer C Taner CE Solmaz U Gezer NS Ozeren M. Prognostic value of fetal thymus size in intrauterine growth restriction. J Ultrasound Med. (2016) 35:511–7. 10.7863/ultra.15.05039 26860482

[13] McDade TW Beck MA Kuzawa CW Adair LS. Prenatal undernutrition and postnatal growth are associated with adolescent thymic function. J Nutr. (2001) 131:1225–31. 10.1093/jn/131.4.1225 11285331

[14] Ballow M Cates KL Rowe JC Goetz C Desbonnet C. Development of the immune system in very low birth weight (less than 1500 g) premature infants: concentrations of plasma immunoglobulins and patterns of infections. Pediatr Res. (1986) 20:899–904. 10.1203/00006450-198609000-00019 3748663

[15] Embleton NE Pang N Cooke RJ. Postnatal malnutrition and growth retardation: an inevitable consequence of current recommendations in preterm infants? Pediatrics. (2001) 107:270–3. 10.1542/peds.107.2.270 11158457

[16] Vlaardingerbroek H van Goudoever JB van den Akker CHP. Initial nutritional management of the preterm infant. Early Hum Dev. (2009) 85:691–5. 10.1016/j.earlhumdev.2009.08.052 19762174

[17] Hsiao C-C Tsai M-L Chen C-C Lin H-C. Early optimal nutrition improves neurodevelopmental outcomes for very preterm infants. Nutr Rev. (2014) 72:532–40. 10.1111/nure.12110 24938866

[18] Ortiz-Espejo M Pérez-Navero JL Olza-Meneses J Muñoz-Villanueva MC Aguilera-García CM Gil-Campos M. Prepubertal children with a history of extra-uterine growth restriction exhibit low-grade inflammation. Br J Nutr. (2014) 112:338–46. 10.1017/S0007114514000920 24832925

[19] Rytter MJH Kolte L Briend A Friis H Christensen VB The immune system in children with malnutrition—a systematic review. PLoS ONE. (2014) 9:e105017 10.1371/journal.pone.0105017 25153531 PMC4143239

[20] Clark RH Thomas P Peabody J. Extrauterine growth restriction remains a serious problem in prematurely born neonates. Pediatrics. (2003) 111:986–90. 10.1542/peds.111.5.986 12728076

[21] Ehrenkranz RA Younes N Lemons JA Fanaroff AA Donovan EF Wright LL. Longitudinal growth of hospitalized very low birth weight infants. Pediatrics. (1999) 104:280–9. 10.1542/peds.104.2.280 10429008

[22] Han F Hu L Xuan Y Ding X Luo Y Bai S. Effects of high nutrient intake on the growth performance, intestinal morphology and immune function of neonatal intra-uterine growth-retarded pigs. Br J Nutr. (2013) 110:1819–27. 10.1017/S0007114513001232 23596997

[23] Hu L Liu Y Yan C Peng X Xu Q Xuan Y. Postnatal nutritional restriction affects growth and immune function of piglets with intra-uterine growth restriction. Br J Nutr. (2015) 114:53–62. 10.1017/S0007114515001579 26059215

[24] Che L Thymann T Bering SB Le Huërou-Luron I D'Inca R Zhang K. IUGR does not predispose to necrotizing enterocolitis or compromise postnatal intestinal adaptation in preterm pigs. Pediatr Res. (2010) 67:54–9. 10.1203/PDR.0b013e3181c1b15e 19816236

[25] D'Inca R Gras-Le Guen C Che L Sangild PT Le Huërou-Luron I. Intrauterine growth restriction delays feeding-induced gut adaptation in term newborn pigs. Neonatology. (2011) 99:208–16. 10.1159/000314919 20881437

[26] Nguyen DN Jiang P Frøkiær H Heegaard PMH Thymann T Sangild PT. Delayed development of systemic immunity in preterm pigs as a model for preterm infants. Sci Rep. (2016) 6:36816. 10.1038/srep36816 27830761 PMC5103294

[27] Mærkedahl RB Frøkiær H Lauritzen L Metzdorff SB. Evaluation of a low-cost procedure for sampling, long-term storage, and extraction of RNA from blood for qPCR analyses. Clin Chem Lab Med. (2015) 53:1181–8. 10.1515/cclm-2014-1054 25720080

[28] Levy O. Innate immunity of the newborn: basic mechanisms and clinical correlates. Nat Rev Immunol. (2007) 7:379–90. 10.1038/nri2075 17457344

[29] Angelone DF Wessels MR Coughlin M Suter EE Valentini P Kalish LA. Innate immunity of the human newborn is polarized toward a high ratio of IL-6/TNF-alpha production *in vitro* and *in vivo*. Pediatr Res. (2006) 60:205–9. 10.1203/01.pdr.0000228319.10481.ea 16864705

[30] Radmacher PG Looney SW Rafail ST Adamkin DH. Prediction of extrauterine growth retardation (EUGR) in VVLBW infants. J Perinatol. (2003) 23:392–5. 10.1038/sj.jp.7210947 12847535

[31] Grazia Roncarolo M Gregori S Battaglia M Bacchetta R Fleischhauer K Levings MK Interleukin-10-secreting type 1 regulatory T cells in rodents and humans. Immunol Rev. (2006) 212:28–50. 10.1111/j.0105-2896.2006.00420.x 16903904

[32] Siewe L Bollati–Fogolin M Wickenhauser C Krieg T Müller W Roers A Interleukin-10 derived from macrophages and/or neutrophils regulates the inflammatory response to LPS but not the response to CpG DNA. Eur J Immunol. (2006) 36:3248–55. 10.1002/eji.200636012 17111348

[33] Akbari O DeKruyff RH Umetsu DT. Pulmonary dendritic cells producing IL-10 mediate tolerance induced by respiratory exposure to antigen. Nat Immunol. (2001) 2:725–31. 10.1038/90667 11477409

[34] Leung KS Fung KP Sher AH Li CK Lee KM. Plasma bone-specific alkaline phosphatase as an indicator of osteoblastic activity. J Bone Joint Surg Br. (1993) 75:288–92. 10.1302/0301-620X.75B2.8444951 8444951

[35] Drifte G Dunn-Siegrist I Tissières P Pugin J. Innate immune functions of immature neutrophils in patients with sepsis and severe systemic inflammatory response syndrome^*^. Crit Care Med. (2013) 41:820–32. 10.1097/CCM.0b013e318274647d 23348516

[36] Taneja R Sharma AP Hallett MB Findlay GP Morris MR. Immature circulating neutrophils in sepsis have impaired phagocytosis and calcium signaling. Shock. (2008) 30:618–22. 10.1097/SHK.0b013e318173ef9c 18496237

[37] Lieschke G Grail D Hodgson G Metcalf D Stanley E Cheers C. Mice lacking granulocyte colony-stimulating factor have chronic neutropenia, granulocyte and macrophage progenitor cell deficiency, and impaired neutrophil mobilization. Blood. (1994) 84:1737–46. 7521686

[38] Furze RC Rankin SM. Neutrophil mobilization and clearance in the bone marrow. Immunology. (2008) 125:281–8. 10.1111/j.1365-2567.2008.02950.x 19128361 PMC2669132

