# Supplementary material for: Growth Restriction and Systemic Immune Development in Preterm Piglets
Source: Front Immunol. 2019 Oct 10;10:2402. doi: 10.3389/fimmu.2019.02402 (PMC6795705; doi:10.3389/fimmu.2019.02402)
Supplement: Supplementary file 1 [file Table_1.pdf]

Supplementary table 1: Number of censored samples for each gene

Gene	Day 8	Day 19
TBET	**1/38**	**1/37**
	F-GR: 0/1	F-GR: 1/1
	F-CON: 1/1	F-CON: 0/1
	P-GR: 0/1	P-GR: 0/1
	P-CON: 1/1	P-CON: 1/1
TNFA	**0/38**	**1/37**
		F-GR: 0/1
		F-CON: 1/1
		P-GR: 0/1
		P-CON: 1/1
IL2	**6/38**	**4/37**
	F-GR: 1/6	F-GR: 0/4
	F-CON 5/6	F-CON: 4/4
	P-GR: 1/6	P-GR: 2/4
	P-CON: 5/6	P-CON: 2/4
IFNG	**5/38**	**3/37**
	F-GR: 1/6	F-GR: 1/3
	F-CON 5/6	F-CON: 2/3
	P-GR: 0/6	P-GR: 1/3
	P-CON: 6/6	P-CON: 2/3
IL4	**0/38**	**0/37**
IL6	**0/38**	**0/37**
IL10	**0/38**	**4/37**
		F-GR: 1/4
		F-CON: 3/4
		P-GR: 1/4
		P-CON: 3/4
GATA3	**7/38**	**2/37**
	F-GR: 0/7	F-GR: 0/2
	F-CON: 7/7	F-CON: 2/2
	P-GR: 1/7	P-GR: 0/2
	P-CON: 6/7	P-CON: 2/2

F-GR: Fetal growth restricted group, F-CON: Fetal growth restricted control group, P-GR: Postnatal growth restricted group, P-CON: Postnatal growth restricted control group
